# Effects of the Thai massage program on range of motion of lower extremities and vertical jump performance in collegiate volleyball players, Burapha Univeristy, Thailand

**DOI:** 10.1186/1757-1146-7-S1-A44

**Published:** 2014-04-08

**Authors:** Sirikool Klumkool, Kawiya Sintara, Sakesan Tongkhambancsong

**Affiliations:** 1 Faculty of Sport Science, Burapha University, Thailand; 2 Faculty of Education, Burapha University, Thailand; 3 School of Health Science, Mae Fah Luang University, Thailand

## 

Sport massage can enhance athletic physical performance which is similar to Thai traditional massage but no evidence reports the increase in athletic performance before competition. The purpose of this research was to study effects of the Thai massage program on range of motion (ROM) of lower extremities and vertical jump performance (VJP) in collegiate volleyball players. Twelve males and twelve females in collegiate volleyball, age between 18-22 years, were randomly divided into two groups; experimental (N = 12) and control groups (N=12). All subjects were measured ROM including knee flexion, ankle plantarflexion and dorsiflexion, and VJP. Thai massage program was applied to the experimental group for 30 minutes and the control group sat still for 30 minutes. The post-test was done and the tests were repeatedly measured every other day for 3 days. The mean differences of the pre and post-test data were calculated and statistically analyzed by using repeatedly measured ANCOVA at the level of .05. The results showed that the mean difference of ROM of Lt. knee flexion, Rt. knee flexion, Lt. ankle plantarflexion, Rt. ankle plantarflexion, Lt. ankle dorsiflexion, and Rt. ankle dorsiflexion were significantly different between groups (*p* = .015, .002, .011, .004, .000, and .000, respectively). Vertical jump performance was significantly different between groups (*p* = .026). Thai massage program was able to increase ROM of the lower extremities and jump performance. Thai Massage was able to warm for improving performance in competition.

